# Prevalence, molecular detection, and virulence gene profiles of *Campylobacter* species in humans and foods of animal origin

**DOI:** 10.14202/vetworld.2020.1430-1438

**Published:** 2020-07-24

**Authors:** Ashraf M. A. Barakat, Khaled A. Abd El-Razik, Hassan A. Elfadaly, Nagwa S. Rabie, Sabry A. S. Sadek, Abdulaziz M. Almuzaini

**Affiliations:** 1 Department of Zoonotic Diseases, National Research Centre, Dokki, Giza, Egypt; 2 Department of Animal Reproduction, National Research Centre, Dokki, Giza, Egypt; 3 Department of Poultry Diseases, National Research Centre, Dokki, Giza, Egypt; 4 Department of Veterinary Medicine, College of Agriculture and Veterinary Medicine, Qassim University, Buraydah, Saudi Arabia

**Keywords:** *Campylobacter*, Egypt, food, human stool, multiplex polymerase chain reaction, virulence genes

## Abstract

**Background and Aim:**

Campylobacteriosis is one of the most well-characterized bacterial foodborne infections worldwide that arise chiefly due to the consumption of foods of animal origin such as poultry, milk, and their products. The disease is caused by numerous species within the genus *Campylobacter*, but *Campylobacter jejuni* is the most commonly isolated species from established cases of human campylobacteriosis. This study was conducted to determine the prevalence and virulence of *Campylobacter* isolates from human, chicken, and milk and milk products in Egypt.

**Materials and Methods:**

A total of 1299 samples (547 chicken intestine and liver, 647 milk and milk products, and 105 human stool) were collected and microbiologically investigated, confirmed by multiplex polymerase chain reaction (PCR) targeting the 23S rRNA, *hipO*, and *glyA* genes specific for *Campylobacter* spp., *C. jejuni*, and *Campylobacter Coli*, respectively, followed by virulence genes (*Campylobacter* adhesion to fibronectin F [*cadF*] and *cdtB*) detection using PCR.

**Results:**

About 38.09%, 37.84%, and 8.5% of human stool, chicken, and milk and milk product samples, respectively, were bacteriologically positive, with a total of 302 *Campylobacter* isolates. All isolates were molecularly confirmed as *Campylobacter* spp. (100%) where 285 isolates (94.37%) were identified as *C. jejuni* and 17 isolates (5.62%) as *C. coli*. Regarding the virulence pattern, all isolates (100%) carried *cadF* gene while cytolethal distending toxin B gene was definite in 284/302 isolates (94%), concisely, 282/285 (98.94%) *C. jejuni* isolates, and in 2/17 (11.76%) *C. coli* isolates.

**Conclusion:**

The widespread presence of these highly virulent *Campylobacter*, especially *C. jejuni*, proofs the urgent need for the implementation of stringent control, public health, and food protection strategies to protect consumers from this zoonotic pathogen. The availability of information about pathogen virulence will enable enhanced local policy drafting by food safety and public health officials.

## Introduction

Campylobacteriosis is a serious zoonotic gastrointestinal disease worldwide, and most cases are mainly caused by *Campylobacter jejuni*. Poultry plays an important role in the transmission of campylobacteriosis to humans [1]. *C. jejuni* colonizes the chicken gut primarily in the cecum and small intestine but also colonizes the liver and spleen [2]. Thus, the intestinal tract of chickens supplies a reservoir of *Campylobacter* that may spread through fecal material at farms or during processing [3]. Human *Campylobacter* infection may be due to either the consumption of undercooked meat or the cross-contamination of ready-to-eat food during preparation or storage [4]. Worldwide, *C. jejuni* is responsible for 85% of foodborne *Campylobacter* enteritis in humans and is the most frequently isolated *Campylobacter* species recovered from poultry, while the remaining cases are primarily attributed to *Campylobacter coli* [5]. *Campylobacter* species are the main cause of bacterial gastrointestinal disease campylobacteriosis, which causes diarrhea, sometimes dysentery syndrome, and cramps, fever, and pain in developing countries. In particular, *C. jejuni* and *C. coli* are accountable for campylobacteriosis [6].

The isolation of campylobacters using the culture method is considered the gold standard for campylobacteriosis disease diagnosis; however, it is time-consuming and laborious because of the fastidious nature of campylobacters [7]. In addition, the differentiation of species using biochemical assays is difficult due to the phylogenetic relatedness of *C. jejuni* and *C. coli* species [8]. Thus, molecular-based assays, such as polymerase chain reaction (PCR) and sequencing, can enable easy, rapid, and specific detection and epidemiological applications [9]. For this purpose, various genes have been used [10].

Several genes have been linked to *Campylobacter* virulence, but the most important are cytolethal distending toxin B (*cdtB*), which disrupts mucosal ­barriers by causing host cell death, *Campylobacter* adhesion to fibronectin F (*cadF*), and the heat survival and stress response proteins *htrB* and *clpP*, which are important for survival [10,11]. The disease severity depends on the virulence of the strain and on the host’s immune condition *. cadF* is one of the reference virulence genes that encode a protein involved in the invasion and adhesion of *C. jejuni* [12], and this gene is present at a high level in *C. jejuni* isolates [13].

Despite the increased recovery of *Campylobacter* as a foodborne pathogen, the specific virulence and pathogenic mechanisms by which microaerophilic *Campylobacter* species cause infection are still poorly understood [14]. The putative virulence factor for adhesion and invasion of epithelial cells, toxin production, and flagellar motility are thought to be important virulence mechanisms [15]. However, different studies have indicated that different virulence markers play a role in the colonization, adherence, and invasion of *Campylobacter* spp. in animals and humans [15].

The aim of this study was to investigate the prevalence of *Campylobacter* spp. using conventional and molecular tools and to determine the virulence gene profile of *Campylobacter* spp. in humans and foods of animal origin in Egypt.

## Materials and Methods

### Ethical approval

All aspects of the study were performed in accordance with national ethics regulations and approved by the National Research Ethical Committee, National Research Centre, Giza, Egypt (Ethical Approval no 16220). Written consent was taken from patients before collecting stool samples.

### Sample collection

A total of 1299 samples (Table-1) from chickens (n=547), milk and milk products (n=647) from various markets, and human stool (n= 105) were collected randomly from different governorates in Egypt (Cairo, Giza, Fayoum, and Qalyubia) from January 2018 to December 2018. The human stool specimens were collected at random from people with diarrhea who were admitted to different laboratories, people in contact with backyard chickens and slaughterhouses and from diarrheic children admitted to hospitals for kids in Egypt. Ten grams of each sample (chicken intestine and liver, milk and milk products, and human stool) were collected in a sterile sample collection vial and transferred to the laboratory. All samples were immediately stored at 4°C and processed to isolate campylobacters.

**Table-1:** Samples collected from different localities in Egypt.

Site of samples	Number of samples	Chicken samples		Milk and milk products			Human stool
	
		Intestine	Liver	Milk	Cheese	Yoghurt	
Giza	400	165	60	77	40	41	17
Fayoum	319	115	10	65	52	47	30
Cairo	266	60	10	65	40	59	32
Qalyubia	314	80	47	67	48	46	26
Total	1299	420	127	274	180	193	105

### Isolation and identification of *Campylobacter*

A loop of each sample was homogenized in sterile thioglycollate broth (Oxoid). Broth samples were incubated at 42°C for 48 h in a microaerobic atmosphere using an anaerobic jar with CampyGen sachets, which generates 10% CO_2_, 5% O_2_, and 85% N_2_. A loopful of enrichment broth was streaked onto mCCDA plates (Oxoid) and incubated under microaerobic condition at 42°C for 48 h followed by microscopic examination to examine their characteristic motility utilizing phase contrast microscope after staining by Gram’s stain presenting seagull appearance. All isolates were subjected to biochemical tests, such as catalase, oxidase, urease, nitrate reduction, indole acetate hydrolysis, and hippurate hydrolysis tests and susceptibility tests to cephalothin and nalidixic acid by the disk diffusion method [16].

### Molecular characterization of *Campylobacter* species

#### DNA extraction

DNA was extracted by the heating and snap chilling method [17]. Two to three colonies of fresh bacterial growth were collected from culture medium, suspended in nuclease-free demonized water, and heated at 95°C for 10 min. The samples were cooled immediately and centrifuged for 5 min at room temperature. The supernatant was separated and 3 μl was used as the DNA template.

#### Confirmation of Campylobacter spp., C. jejuni, and C. coli isolates by multiplex PCR

A multiplex PCR reaction was used for the confirmation of biochemically identified *Campylobacter* spp. through targeting *23S* *rRNA* specific for *Campylobacter* spp., *hipO* gene specific for *C. jejuni*, and *glyA* gene specific for *C. coli* (Table-2) [18-21]. Primers were utilized in a 25 μl reaction containing 12.5 μl of 2× ViRed Taq Master Mixture (Cat. no. CLMM01, Vivantis Technologies, Malaysia), 1 μl of each primer (20 pmol), 5.5 μl of water, and 3 μl of template. Cycling conditions begin with initial denaturation at 95°C for 6 min, followed by 35 cycles of denaturation at 95°C for 30 s, annealing at 59°C for 30 s, and extension at 72°C for 30 s with a single final extension step at 72°C for 10 min. The PCR products were separated by electrophoresis on 1% agarose gel.

**Table-2:** Primer sets for PCR amplification of the four target genes of *Campylobacter* species.

Target gene	Primer	Primer sequence (5’→’)	Size (in bp)	References
*Campylobacter* spp. *23S rRNA*	CB1 CB2	TATACCGGTAAGGAGTGCTGGAG ATCAATTAACC TTCGAGCACCG	650	[19]
*Campylobacter jejuni hipO*	CJF CJR	ACTTCTTTATTGCTTGCTGC GCCACAACAAGTAAAGAAGC	323	[19]
*Campylobacter coli glyA*	CCF CCR	GTAAAACCAAAGCTTATCGTG TCCAGCAATGTGTGCAATG	126	[18]
*Campylobacter* adherence gene (*cadF*)	cad F cad R	TTGAAGGTAATTTAGATATG CTAATACCTAAAGTTGAAAC	400	[20]
Cytolethal distending toxin subunit B gene (*cdtB*)	CdtB-F CdtB-R	GTTGGCACTTGGAATTTGCAAGGC GTTAAAATCCCCTGCTATCAACCA	495	[21]

PCR=Polymerase chain reaction

### Virulence gene characterization of *Campylobacter* isolates

The confirmed isolates of *Campylobacter* species were characterized for *in vitro* detection of virulence genes by PCR for two well-known virulence genes encoding the *cadF* [20] and *cdtB* genes [14]. The details of the primers for the target virulence genes are described in Table-2. Cycling conditions were as previous with annealing at 45^°^C for *cadF* gene and 57°C for *cdtB* gene.

### Phylogenetic tree construction

The positive PCR products targeting the *cadF* gene of two *C. jejuni* samples (CJ1 and CJ2) were sequenced by MACROGEN Company (Korea) on 3730XL sequencers (Applied Biosystems, USA). The accuracy of the data was confirmed by two-directional sequencing with the forward and reverse primers used in PCR. The nucleotide sequences obtained in this study were analyzed using the programs BioEdit 7.0.4.1 and Muscle (https://www.ebi.ac.uk/Tools/msa/muscle/). The resulting sequences were aligned with the *cadF* virulence gene of *Campylobacter* spp. reference sequences (Table-3) using neighbor-joining analysis of the aligned sequences implemented in the program CLC Genomics Workbench 3.

**Table-3:** Details of *Campylobacter jejuni* isolates used in the present study and available in GenBank.

S. No.	Organism	Strain	Host	Isolation source	Country	Access. No.
1	*Campylobacter jejuni*	CJ1	Broiler chicken	Intestine	Egypt	MN103381
2	*Campylobacter jejuni*	CJ2	Broiler chicken	Liver	Egypt	MN103382
3	*Campylobacter jejuni* subsp *. jejuni*	D42a	Chicken	Caecum	USA	CP007751
4	*Campylobacter jejuni*	RM1285	Chicken	Breast exudate	USA	CP012696
5	*Campylobacter jejuni*	YQ2210	Turkey	----	USA	CP017859
6	*Campylobacter jejuni*	104	Chicken	----	Brazil	CP023343
7	*Campylobacter jejuni*	CFSAN032806	Chicken	Breast	USA	CP023543
8	*Campylobacter jejuni*	FDAARGOS_421	Chicken	Carcass	USA	CP023866
9	*Campylobacter jejuni*	NCTC 12664	Chicken	----	United Kingdom	CP028912
10	*Campylobacter jejuni*	FORC_083	Chicken	Meat	South Korea	CP028933
11	*Campylobacter jejuni* subsp *. jejuni*	CLB104	Chicken	Liver	United Kingdom	CP034393
12	*Campylobacter jejuni* subsp *. jejuni*	00-2425	Human	Stool	Canada	CP006729
13	*Campylobacter jejuni*	CJ074CC443	Human	----	Finland	CP012216
14	*Campylobacter jejuni* subsp *. jejuni*	RM3196	Human	----	South Africa	CP012690
15	*Campylobacter jejuni*	FDAARGOS_263	Human	Stool	USA	CP022077
16	*Campylobacter jejuni*	FDAARGOS_422	Human	----	USA	CP023867
17	*Campylobacter jejuni* subsp *. jejuni*	huA17	Human	Stool	Germany	CP028372
18	*Campylobacter jejuni* subsp *. jejuni*	NCTC10983	Human	Blood	United Kingdom	LR134511

## Results

### Identification of *Campylobacter* species

In this investigation, samples were collected from Giza, Fayoum, Cairo, and Qalyubia in Egypt for the isolation of *Campylobacter* spp. from chicken, milk, milk products, and human stool. *Campylobacter* spp. were isolated from 37.84% of chickens, 8.5% of milk and milk product samples, and 38.09% of human stool samples with a total of 302 (23.24%) *Campylobacter* spp. isolates (Table-4).

**Table-4:** Prevalence of *Campylobacter* genus in the examined samples by conventional and molecular methods.

Type of samples	Incidence by conventional method		Incidence of *C. jejuni* by multiplex PCR		Incidence of *C. coli* by multiplex PCR	
		
	n	%	n	%	n	%
Chickens	207/547	37.84	193/207	93.23	14/207	6.76
Intestine	160/420	38.09	158/160	98.75	2/160	1.25
Liver	47/127	37	35/47	74.46	12/47	25.5
Milk and milk products	55/647	8.5	55/55	100	0/55	0
Raw milk	14/274	5.1	14/14	100	0/14	0
Cheese	14/180	7.77	14/14	100	0/14	0
Yoghurt	27/193	13.98	27/27	100	0/27	0
Human stool	40/105	38.09	37/40	92.5	3/40	7.5
Total	302/1299	23.24	285/302	94.37	17/302	5.62

*C. jejuni=Campylobacter jejuni, C. coli= Campylobacter coli,* PCR=Polymerase chain reaction

### Confirmation of *Campylobacter* spp., *C. jejuni*, and *C. coli* isolates by multiplex PCR

**Figure-1:**
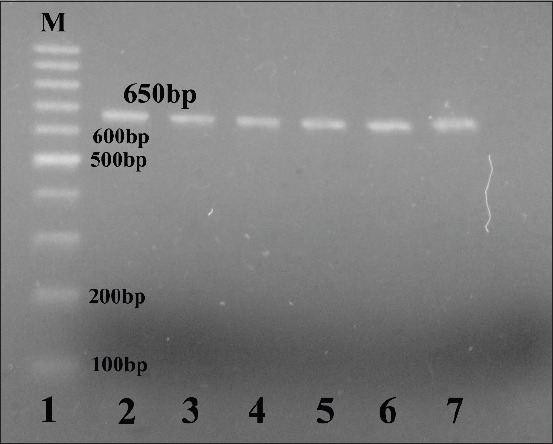
Amplification of the *23S rRNA* gene of *Campylobacter* spp. positive amplification appeared at 650 bp, lane 1: 100 bp ladder, lane 2: The positive control, lanes 3-7: Positive for *Campylobacter* spp.

**Figure-2:**
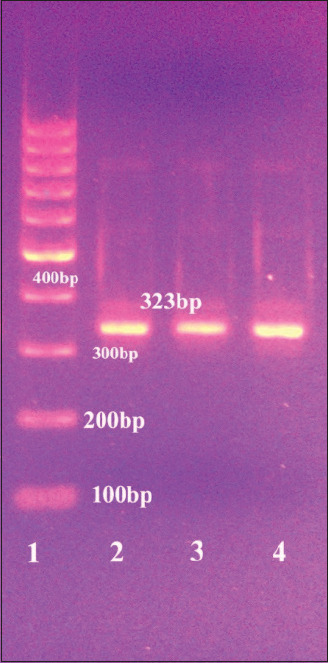
Amplification of the *hipO* gene of *Campylobacter jejuni*, positive amplification appeared at 323 bp, lane 1: 100 bp ladder, lane 2: The positive control *C. jejuni*, lanes 3 and 4: Positive for *C. jejuni*.

**Figure-3:**
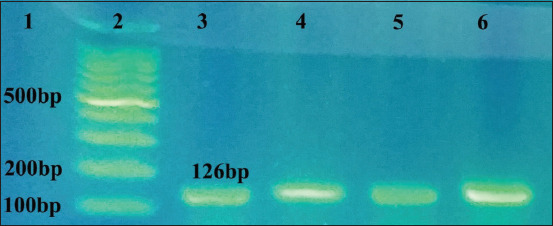
Amplification of the *glyA* gene of *Campylobacter coli*, positive amplification appeared at 126 bp, lane 1: 100 bp ladder and lane 2: The positive control *C. coli*, lanes 4-6: Positive for *C. coli*.

The 302 biochemically identified *Campylobacter* isolates were molecularly confirmed by the amplification of *23S rRNA* gene of *Campylobacter* spp., the *hipO* gene specific to *C. jejuni*, and the *glyA* gene specific to *C. coli*. All the 302 isolates were confirmed as *Campylobacter* (Figure-1), of which 94.37% as C *. jejuni* and 5.62% as *C. coli* (Figures-2 and 3, Table-4).

In detail, 69.35%, 57.5%, and 63.33% of isolates from chicken, milk and milk product, and human stool samples were confirmed as *C. jejuni* while 6.76% and 5.62 of chicken and human stools samples as *C. coli*, respectively (Table-4).

### Virulence determinants

**Figure-4:**
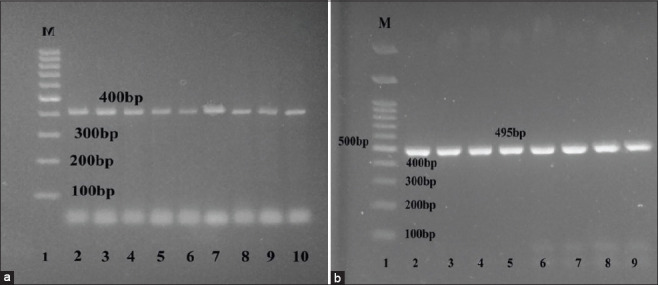
Agarose gel electtrophoresis of polymerase chain reaction products obtained with primers for, (a) the *cadF* gene. Lane 1, 100 bp ladder; 2-10, 400 bp fragment of the *cadF* gene of *Campylobacter jejuni* and *Campylobacter coli*. (b) The *cdtB* gene. Lane 1, 100 bp ladder; 2-9, 495-bp fragment of the *cdtB* gene of *C. jejuni* and *C. coli*.

With regard to the virulence pattern, all isolates carried the virulence associate gene *cadF* (100%), while *cdtB* gene was positive in 284 out of the 302 isolates (94%). Briefly, 282 out of 285 (98.94%) of *C. jejuni* isolates and 2 out of 17 (11.76%) of *C. coli* isolates were positive for *cdtB gene* (Figure-4a and b).

### Nucleotide sequence accession numbers

**Figure-5:**
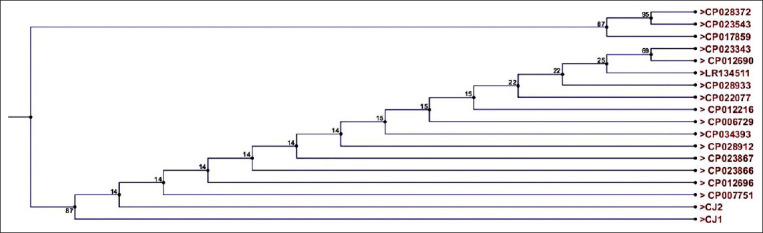
Phylogenetic relationship of selected strains of *Campylobacter jejuni* from poultry, milk and milk products, and human; the accession numbers of the isolates used are given.

Two sequences (*C. jejuni cadF* gene) used in this study (CJ1 and CJ2) have been deposited in the GenBank database under the accession nos. MN103381-MN103382. Phylogenetic analysis confirmed that the two isolates were *C. jejuni* with homology results of 99-100%. In the phylogenetic tree, all Egyptian isolates formed two separate clusters (Figure-5). Phylogenetic analysis showed that CJ1 (MN103381) and CJ2 (MN103382), which were isolated from the intestine and liver of chickens, respectively, had typical homology with *C. jejuni* isolated from either chicken or human (Figure-5).

## Discussion

In the current study, *Campylobacter* spp. were isolated from 37% to 38.09% of the liver and intestine of chickens, respectively. Similar isolation rate (28.3%) from cloacal swabs was reported in Egypt [22]. Comparable isolation rates (31.9% and 39.2%) were reported in Vietnam [23] and Estonia [24], respectively. Reduced isolation rate (16.83%) was reported by Abushahba *et al*. [25]. The reported prevalence rate of *Campylobacter* spp. is higher in the previous studies than in this study, for instance, 41.2% and 48.5% in Egypt [22,26] and 58% in Brazil [27]. The aforementioned high isolation rates could be attributed to the extensive type of chicken management that increases the exposure of birds to *Campylobacter* infection through insects, rodents, contaminated water, and poor housing hygiene [28]. In addition, high isolation rates of 82.9% in Italy and 51.5% in Nigeria from chicken cloacal swabs, respectively, were reported [29,30], which could be a result of using only conventional methods.

The variation in the isolation rate of *Campylobacter* spp. between different studies could be attributed to the age of the examined chickens [31] and the difference in the sanitation levels, while handling and processing chickens, the type and site of the examined samples, the sampling season, the laboratory methodologies employed for isolation, husbandry and management, and the production system have the greatest impact on the prevalence rate of campylobacters [32].

Cattle play an important role in human campylobacteriosis. There are cattle-related outbreaks that indicate that raw milk and dairy products are the second most frequent sources of infection. Direct contamination of milk may occur through feces or as a consequence of mastitis [33]. In the current study, *Campylobacter* spp. was isolated from 8.5% of milk and milk products samples (5.1%, 7.77%, and 13.98% of raw milk, cheese, and yoghurt samples, respectively). The higher percentage of *Campylobacter* in milk products than in raw milk is explained by the contamination of milk products because of unhygienic conditions during the preparation of milk products. A similar isolation rate of *Campylobacter* from raw milk (7.2%) was reported in Turkey [1]. Relatively low prevalence rates of 2.91% and 2.32% were reported [17,34], respectively. In contrast, high prevalence rates of *Campylobacter* in milk at 17.2%, 20%, and 66.8% were reported [35-37]. Here, *Campylobacter* spp. were isolated from 7.77% and 13.98% of cheese and yoghurt samples, respectively. Similarly, a prevalence rate of 5.0% from cheese was reported by Giacometti *et al*. [38]. In the contrary, *Campylobacter* was absent from milk product samples in some reports [17,39]. This emphasizes the importance of milk and dairy products as a potential source of *Campylobacter*.

In general, *Campylobacter* is the most common bacterium that induces gastroenteritis in humans globally and can be fatal to young children, geriatric patients, and immunocompromised patients [40]. In the current study, an isolation rate of 38.09% was reported in stool samples from humans. These results were concurrent with the isolation rate (33.33%) detected by Rouby *et al*. [41] in Egypt but were higher than those (27.5%) detected by Abushahba *et al*. [25] in Egypt. The high percentage detected in our study could be attributed to the inclusion of stool samples primarily obtained from gastroenteritis-infected individuals rather than investigating the disease in the general population.

PCR is still rapid, specific, sensitive, and of substantial interest for the recognition and verification of *Campylobacter* species. Thus, PCR is a dependable substitute for conventional culture [3,42]. Molecular methods were used to confirm the detected *Campylobacter* species and to differentiate between *C. jejuni* and *C. coli*, as the discrimination of *C. jejuni* and *C. coli* is considered difficult because it depends only on a single phenotypic test [8]. Therefore, here, multiplex PCR was used to identify the isolated *Campylobacter* spp. (302 isolates) by targeting the *23S rRNA* specific for *Campylobacter* spp., *hipO* gene of *C. jejuni*, and the *glyA* gene of *C. coli*. The multiplex PCR results confirmed all the 302 biochemically identified isolates as *Campylobacter* spp. of which 285 isolates as *C. jejuni* (94.37%) and 5.62% as *C. coli*. A total percentage of 69.35%, 57.5%, and 63.33% of isolates from chicken, milk and milk product, and human stool samples were confirmed as *C. jejuni* while 6.76% and 5.62 of chicken and human stools samples as *C. coli*, respectively.

One hundred and ninety-three out of 207 *Campylobacter* isolates (93.23%) recovered from chicken samples were identified as *C. jejuni* using multiplex PCR. Similar results using PCR, where *C. jejuni* was the predominant isolate and reached prevalence rates as high as 87.5%, 90%, and 89% in frozen chicken carcasses as reported in Egypt [3], in Great Britain [43], and in Vietnam [23], respectively. The predominance of *C. jejuni* may be due to its ability to survive high and low temperatures, low pH, and dry conditions [44]. This was contrary to the findings in Egypt [22,26] and in Argentina [45], those reported *C. jejuni* as the only species isolated and molecularly identified from chickens. Moreover, 14 out of 207 *Campylobacter* isolates (6.76%) recovered from chicken samples were identified as *C. coli* using multiplex PCR. Only a few reports are available regarding *C. coli* as the predominant isolate in Egypt [42] and Greece [46]. This may be due to the type of feed ration because *C. jejuni* does not frequently colonize birds receiving plant protein-based feed [46].

A total of 55 *Campylobacter* isolates recovered from milk and milk products were identified as *C. jejuni* (100%) using multiplex PCR. This was concurrent with the findings in Egypt [17], indicating that this species is distributed widely in the study areas. Similar results reported that *C. jejuni* was the predominant isolate (85.7%) in Turkey [1].

A total of 37 out of 40 *Campylobacter* isolates recovered from human stool were identified as *C. jejuni* (92.5%) and 3 out of 40 isolates as *C. coli* (7.5%) using multiplex PCR. The isolation rates of *C. jejuni* that were higher than those of *C. coli* from *Campylobacter*-positive stool samples were in relative agreement with that (61.7%) of Sainato *et al*. [40] and with higher prevalence than those (27.5% and 8.4%) of Abushahba *et al*. [25] and Omara *et al*. [47] in Egypt, respectively. The high prevalence observed in our study may be a result of collecting samples from humans originating from villages, where basic hygienic standards and precautions for the contact and handling of live poultry are usually not adopted. This was in contrast with the findings of Rouby *et al*. [41] who reported that PCR revealed that all isolates were *C. jejuni*. Moreover, *C. coli* was the predominant isolate, as high as 1.11% and 3%, as reported in Egypt [25] and in Poland [48], respectively.

The minimal infective dose of *C. jejuni* is very low, which indicates that *C. jejuni* is highly virulent, and a very small number of bacterial cells could cause infection in humans. The virulence of *Campylobacter* species is associated with flagellar motility, adhesion, invasion, and production of cytolethal distending toxins [49]. Several genes have been linked to *Campylobacter* virulence that might contribute to human infection and colonization of chickens [13]. The most important are *cdtB*, which disrupts mucosal barriers by causing host cell death, and *cadF* [26]. The detection of the *cadF* gene in all *C. jejuni* and *C. coli* isolates (100%) from chickens was in agreement with the findings from the previous reports [50,51]; similarly, the results of this study were consistent with the previous results regarding the presence of *C. coli* from humans [51]. However, a higher percentage was identified in this study than in other reports (41.6% and 8%), among all *C. jejuni* isolates [1,50]. The high prevalence rate (100%) of the *cadF* gene in the present study shows that many strains originating from poultry, milk and milk products have potential pathogenic properties toward humans, as reported by Frasao *et al*. [13] and Kalantar *et al*. [19].

According to Gonzalez-Hein *et al*. [52], *CdtB* cytotoxin subunit is encoded by *cdtB* gene that plays an essential role in exerting a toxic effect on cells. In the present study, *Campylobacter* cytotoxic factor (*cdtB*) was confirmed in 98.94% of *C. jejuni* isolates and 11.76% of *C. coli* isolates. The occurrence of *cdtB* from chicken isolates in this study was higher than that of Abu-Madi *et al*. [50] and ELSayed *et al*. [26] who reported 6.7% and 0%, respectively, but less than that of Modi *et al*. [17] that reported the presence of *cdtB* gene in all isolates from bovine and swine tissue. Our findings confirm the relatively higher prevalence of *cdtB* gene in *C. jejuni* in comparison to *C. coli*. This is in parallel with that of Wysok *et al*. [53].

## Conclusion

Our study demonstrated the widespread existence of highly virulent *Campylobacter* isolates, especially *C. jejuni* in chicken, milk and milk product, and human, confirming that this species is a serious infection hazard and public health concern. Moreover, this study emphasizes the urgent need for the implementation of stringent control, public health, and food protection strategies to protect consumers from this zoonotic pathogen. The availability of information about pathogen virulence will enable enhanced local policy drafting by food safety and public health officials and will increase the awareness of this medically and economically important pathogen.

## Authors’ Contributions

AMAB and NSR designed the study, conducted the experiments, and revised the manuscript; HAE and SASS conducted bacterial isolation and identification. KAAE and AMA performed the molecular identification and characterization and wrote the manuscript. All authors read and approved the final manuscript for publication.
